# Evaluation of Real-Time RT-PCR for Diagnostic Use in Detection of Puumala Virus

**DOI:** 10.3390/v11070661

**Published:** 2019-07-19

**Authors:** Silja Niskanen, Anne Jääskeläinen, Olli Vapalahti, Tarja Sironen

**Affiliations:** 1 Department of Virology, University of Helsinki, 00290 Helsinki, Finland; 2 Department of Virology and Immunology, Helsinki University Hospital Laboratory (HUSLAB), 00290 Helsinki, Finland; 3 Department of Veterinary Microbiology and Epidemiology, Faculty of Veterinary Medicine, University of Helsinki, 00290 Helsinki, Finland

**Keywords:** Puumala virus, real-time qRT-PCR, nephropathia epidemica

## Abstract

Puumala virus (PUUV) is the most common cause of hantavirus infection in Europe, with thousands of cases occurring particularly in Northern, Central and Eastern Europe and Russia. It causes a mild form of hemorrhagic fever with renal syndrome also known as nephropathia epidemica (NE) with clinical picture ranging from mild to severe. Currently, the laboratory diagnosis of NE is mainly based on serology. Here, we evaluated a real-time one-step qRT-PCR (PUUV-qRT-PCR) for detection of PUUV with 238 consecutive diagnostic serum samples from patients with suspected PUUV infection. The PUUV-qRT-PCR was both specific and sensitive for PUUV RNA. The analytical sensitivity (limit of detection) was estimated to be four copies of PUUV per reaction. Altogether 28 out of 30 (93%) PUUV IgM positive samples were positive also for PUUV RNA. No false positives were detected and the specificity was thus 100%. Interestingly, one sample was found positive in PUUV-qRT-PCR prior to subsequent IgM and IgG seroconversion. PUUV-qRT-PCR could be used for diagnostics in the early phase of NE infection and might be helpful especially in the rare severe cases when the patient’s condition may deteriorate rapidly.

## 1. Introduction

Puumala virus (PUUV) is a member of the orthohantavirus genus of the *Hantaviridae* family. Hantaviruses cause two types of clinical outcomes, hemorrhagic fever with renal syndrome (HFRS) in Europe and Asia and hantavirus (cardio)pulmonary syndrome (HCPS) on the American continent. The currently known pathogenic hantaviruses are carried by rodents and transmitted to humans by aerosolized excreta of an infected animal with an incubation period of two to four weeks. In addition, several newly found hantaviruses are carried by insectivores and bats but their pathogenicity to humans is unknown [1]. One of the pathogenic hantaviruses is PUUV that causes nephropathia epidemica (NE), a mild form of HFRS, which is endemic in Finland, Sweden, Norway, Russia, the Balkans and parts of central Europe. It is carried by bank voles (*Myodes glareolus*) showing large population density fluctuations with human NE epidemics following bank vole peak densities.

There are 560 to 3800 cases of NE diagnosed annually in Finland, but the true incidence is estimated to be six times higher [2]. PUUV seroprevalence in the Finnish population has been reported to be 5%, and even 11% in the endemic eastern Finland. Other hantaviruses have not been detected in Finland so far, except for the apparently non-pathogenic insectivore-borne viruses, such as Seewis, Asikkala and Boginia viruses [3,4,5].

The clinical image can vary from mild to very severe, and the case-fatality ratio is 0.1% [2]. The majority of the infections are subclinical or remain serologically undiagnosed. The infection starts abruptly with fever and headache, followed by abdominal and back pain, myalgia, nausea and oliguria. Visual disturbances occur in one third of patients, and acute myopia is pathognomonic for NE. Increased serum creatinine, thrombocytopenia, leucocytosis, elevated C-reactive protein and proteinuria are typical laboratory findings [6,7]. Severe infection can lead to renal impairment and haemorrhagic manifestations and 5% of hospitalized patients end up in dialysis [8]. Convalescence begins usually by 14 days from the onset of infection [9]. However, full recovery might take several weeks and sometimes patients might suffer from prolonged hypertension even several years afterwards [10,11]. In addition, chronic hormonal deficiencies may also develop [12]. The rare lethal cases have been attributed to pituitary haemorrhage or circulatory shock [13,14]. 

PUUV is a spherical enveloped virus with three negative-stranded RNAs encoding four different proteins. The L segment, M segment and S segment code for RNA-dependent RNA polymerase, glycoproteins G1 and G2 for the viral particle surface and nucleocapsid protein, respectively. The nucleocapsid protein is the major viral component in the infected cell and, together with the glycoproteins, the major target of immune response [9].

Currently the diagnosis of acute PUUV infection in most countries is based on the parallel detection of virus-specific IgM and IgG, or occasionally only IgM, in the serum. Both IgM and IgG antibodies, particularly against the N protein, rise relatively fast and are usually detected already at onset of symptoms and by the time patients seek for medical care [15]. However, in rare cases the antibodies have been negative up to 5 days after the onset of symptoms [16]. Focus reduction neutralization tests are the golden standard for serotyping hantavirus infections [17].

The aim of this study was to evaluate a one-step real-time RT-PCR (PUUV-qRT-PCR), adjusted to strains circulating in Finland, for diagnosing PUUV infection from acute-phase serum samples as compared to serology and a conventional RT-nested PCR.

## 2. Materials and Methods

### 2.1. Clinical Samples

In order to evaluate the sensitivity of the assay, we collected 30 serum samples from acute NE patients taken at different healthcare centers and hospitals in Finland with positive PUUV IgM results in diagnostics at HUSLAB, Helsinki University Hospital. Detailed background information is not available beyond the definition as acute infections. Diagnostic sensitivity was calculated using MedCalc Software (https://www.medcalc.org/calc/diagnostic_test.php; MedCalc Software, Ostend, Belgium). The limit of detection (LOD) was calculated using seven replicates and Probit Analysis (Statistical Package for Social Sciences; IBM, Chicago, IL; SPSS). To evaluate the specificity, a negative panel of 20 serum samples from patients suspected to have PUUV infection, but confirmed PUUV-antibody negative, were analyzed. RNA of Dobrava (DOBV), Saaremaa (SAAV), Hantaan (HTNV), Tula (TULV) and Topografov (TOPV) viruses were tested in both qRT-PCR and RT-nested-PCR to study the specificity of these tests.

All in all, we selected 238 consecutive serum samples routinely taken from patients with suspected PUUV infection at different health care centers and hospitals in Finland for measurement of PUUV IgM and IgG antibodies at HUSLAB, Department of Virology and Immunology, Hospital District of Helsinki and Uusimaa, Finland. All the samples were gathered between December 2012 and February 2013. The sera were stored at −20 °C (from 2 days up to 4 weeks) prior to RNA extraction. No extra procedures were needed to handle the samples in order to detect the RNA (research permit HUSLAB §32 14.6.2013).

### 2.2. Construction of Synthetic Standard PUUV RNA

The analytical sensitivity was defined by making a dilution series from standard RNA, which was constructed from a TZI9R#5 vector to which a full-length PUUV Sotkamo strain S segment had been cloned. The plasmid was linearized with the restriction enzyme SalI and used as a template for RNA in-vitro transcription using Thermo Scientific TranscriptAid T7 High Yield Transcription Kit (Fermentas, Waltham, MA, USA) according to the manufacturer’s protocol (except instead of 1 μL of template DNA, 5 μL was used). The synthesized RNA was incubated in 37 °C for 15 min with DNAse 1 and purified with GeneJet RNA Purification Kit (Fermentas) according to the RNA Cleanup Protocol. The size of the product was confirmed by agarose-gel electrophoresis and the amount of RNA quantified using NanoDrop. 

### 2.3. Primers and Probes

For the real-time RT-PCR, forward primer (5′-TCC TTG AAA AGC TAC TAC GAG AAA AA-3′) and reverse primer (5′-TTC ATG RCG GGT TAT ATC CTC TT-3′) targeting the 5′ end of the S-segment were used. This site was chosen as the beginning of the S segment is the most conserved part of the PUUV genome and, as mentioned, its product, the nucleocapsid protein, is the most abundant viral component in infected cells. The probe (6-FAM-TGG AAT GAG TGA CTT GAC AG-MGB) was labeled at the 5′ end with reporter 6-carboxyfluorescein (FAM) and at the 3′ end with minor groove binder (MGB). 

For the conventional RT-nested PCR, forward primer PUUSF 532 (5′-TCA TTT GAR GAC ATY AAT GGC ATA AG-3′) and reverse primer PUUSR1232 (5′-ACC ATY TCY TTX CCC CAT TCX AAC AT-3′) were used for the RT and first PCR reaction. Forward primer PUUSF799 (5′-CCX GGX ACA CCA GCA CAR GA-3′) and reverse primer PUUSR1106 (5′-GCT GTG CCX ACA GTY TTX GAT GCC AT-3′) were used for the second PCR reaction. The primers have been modified from those previously described [18].

### 2.4. PUUV-qRT-PCR

RNA was extracted from 140 μL of serum with QIAamp Viral RNA Kit (QIAGEN) according to the manufacturer’s Spin Protocol. Reaction mixture was prepared with qScript One-Step Fast MGB qRT-PCR Kit, low ROX (Quanta), containing 5 μL of eluted RNA, 5 μL 4× One-Step Fast MGB Master Mix, 0.45 μM forward primer, 0.45 μM reverse primer, 0.2 μM probe, 1 μL 20× qScript One-Step Fast MGB RT and 6.8 μL water. The assay was carried out using Stratagene Mx3000P QPCR System and was performed as follows: Initiation at 48 °C for 5 min and 90 °C for 30 s, followed by 45 cycles at 95 °C for 3 s and 60 °C for 30 s.

### 2.5. RT-Nested-PCR

RT reaction mixture was prepared using RevertAid H Minus Reverse Transcriptase Kit: 3 Μl of RNA, 1 mM dNTPs mix, 0.25 μM forward primer PUUSF 532, 0.25 μM reverse primer PUUSR 1232, 4 μL of 5× RT buffer, 8 μL of aqua, 0.5 μL RiboLock RNAse inhibitor and 1.5 μL RevertAid reverse transcriptase.

First PCR reaction mixture was prepared using 5 μL of cDNA from RT reaction, 28.5 μL of deionized distilled water (ddw), 5 μL of 10× Taq reaction buffer, 4 mM MgCl_2_, 0.2 µM forward primer PUUSF532, 0.2 µM reverse primer PUUSR1232, 0.2 mM dNTPs mix and 0.5 μL of Taq DNA polymerase (Fermentas).

Second PCR reaction mixture was prepared using 5 μL of cDNA from the first PCR reaction, 28.5 μL of ddw, 5 μL of 10× Taq reaction buffer, 4 mM MgCl_2_, 0.2 µM forward primer PUUSF799, 0.2 µM reverse primer PUUSR1106, 0.2 mM dNTPs mix and 0.5 μL of Taq DNA polymerase (Fermentas).

RT reaction was performed as follows: 60 min at 45 °C and 10 min at 70 °C. First PCR reaction was initiated at 95 °C for 1 min, followed by 40 cycles of 30 s at 95 °C, 30 s at 53 °C and 60 s at 72 °C and finalized at 72 °C for 5 min. The second PCR reaction was initiated at 95 °C for 1 min, followed by 40 cycles of 30 s at 95 °C, 30 s at 59 °C and 60 s at 72 °C, finalized at 72 °C for 5 min.

The second round PCR products were visualized in agarose gels, purified and sequenced at the sequencing unit of Haartman institute, University of Helsinki. Additional PUUV sequences representing different lineages were collected from GenBank, aligned using ClustalX and subjected to phylogenetic analysis using Bayesian MCMC method implemented in BEAST, version 1.7.2 (http://beast.bio.ed.ac.uk/).

### 2.6. Detection of Immunoglobulins

Detection of IgM by a µ-capture IgM test based on recombinant N [19] and IgG by immunofluorescence assay [19] were performed in the accredited diagnostic laboratory of HUSLAB as previously described.

## 3. Results

### 3.1. PUUV-qRT-PCR Is Both Sensitive and Specific

The analytical sensitivity of the assay was determined using quantified PUUV RNA with seven parallel PUUV-qRT-PCR tests in range of 500 to one PUUV RNA copies per PCR-reaction. The LOD was 3.3 copies per reaction (C_95_ value, copies detectable 95% of the time; SPSS).

Altogether, 30 PUUV IgM positive serum samples from patients with acute NE were stored a maximum of a few weeks in −20 °C in order to evaluate the diagnostic sensitivity (probability that the assay will be positive when the disease is present) of the assay. Out of 30 acute NE cases, 28 were found positive in PUUV-qRT-PCR (Table 1). The diagnostic sensitivity was 93.3% (95% CI: 77.93% to 99.18%; MedCalc). The 20 serum samples from patients negative for PUUV antibodies were all negative in the PUUV-qRT-PCR assay showing specificity of 100%. In addition, samples with RNA of DOBV, SAAV, HTNV, TULV and TOPV hantaviruses were also negative in the PUUV-qRT-PCR assay. No false-positive results were detected. 

Of the 208 serum samples from patients with suspicion of NE infection, but negative for IgM antibodies, one PUUV-IgM negative sample was positive in the PUUV-qRT-PCR. A follow-up sample arrived five days later from the patient and this time the serum sample was again positive in PUUV-qRT-PCR, but had also seroconverted to PUUV, indicating acute NE. 

Of all 30 PUUV IgM positive patient samples, 28 were also positive for PUUV RNA. The two patients with negative result in PUUV-qRT-PCR were further studied from the HUSLAB patient database. These two patients were in late phase of the illness, one to two weeks after the onset of symptoms, and most probably the viremia had passed. 

### 3.2. RT-Nested-PCR and Subsequent Sequencing Shows the High Variability of PUUV Strains Detected

**Figure 1:**
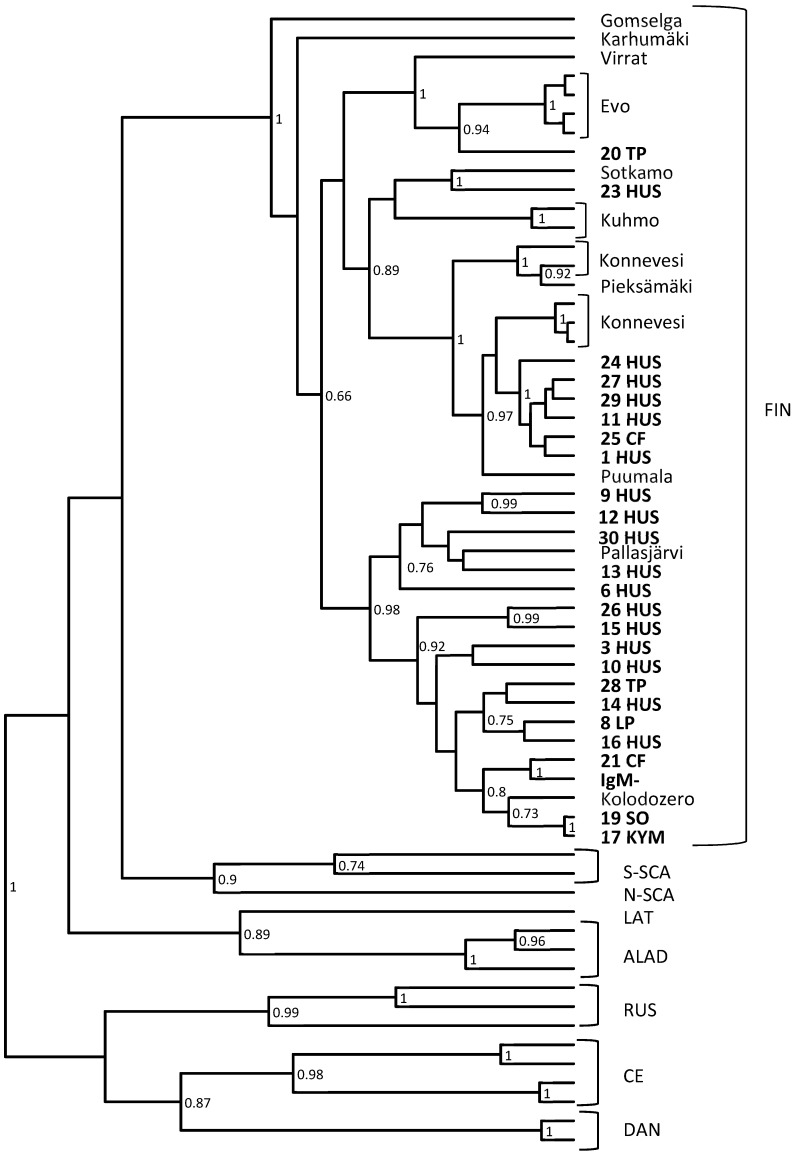
A phylogenetic tree based on partial PUUV S segment sequences. The sequences obtained in this study are shown as bold numbers. In addition to the Finnish (FIN) PUUV strains, we used representative sequences of the northern-Scandinavian (N-SCA), southern-Scandinavian (S-SCA), Latvian (LAT), Russian (RUS), Alpe-Adrian (ALAD) and central European (CE) lineages of PUUV.

All the samples positive for PUUV RNA (*n* = 28) in PUUV-qRT-PCR were further analyzed with conventional RT-nested-PCR followed by sequencing. In addition, 20 negative samples (the specificity panel) were studied by this assay and confirmed negative, as were also RNA representing other hantavirus species (DOBV, SAAV, HTNV, TULV and TOPV). From the PUUV-qRT-PCR positive samples, 25 out of 28 samples were positive also in RT-nested-PCR, and these PCR-products were sequenced and further analyzed (Figure 1). The phylogenetic analysis shows that the assay can detect a wide range of PUUV strains circulating in Finland.

## 4. Discussion

Serology has been the golden standard for hantavirus diagnostics. An alternative could be nucleic acid detection, and we have evaluated here the usefulness of PUUV-qRT-PCR for laboratory diagnostics. The challenge of developing a robust RT-PCR method for routine PUUV diagnostics is the high genomic variance between different strains of PUUV. In this study, we attempted to develop a method that would pick up all variants circulating in Finland. 

RT-PCR has been previously tested for northern Swedish PUUV strains [20]. One hundred samples with clinically suspected NE were studied. In the study, altogether 44 of the patients gave a clearly positive result for PUUV IgM, seven patients gave a weakly positive result and 49 gave a negative result. Of the 44 clearly IgM positive patients, 37 were positive for PUUV RNA and seven were negative. The seven negative samples all originated from southern Sweden, and it was concluded that the method was specific for northern strains. Another study published in 2016 [21] in Sweden demonstrated detection of PUUV RNA in 87.7% of the serologically confirmed cases and in 98.7% of the patients tested within 8 days from the onset. The primers and probes were designed to match a well conserved region in the S segment in order to reach sensitivity for both northern and southern strains. These results are concordant with our study, in which we found a 93.3% diagnostic sensitivity for our assay.

In this study, Puumala virus specific PUUV-qRT-PCR was found to be as sensitive as serology in PUUV diagnostics during the first week of illness. If we sum up all PUUV-positives by either of the methods, the sensitivity is 30/31 (96.77%) for antibodies and 29/31 (93.54%) for RNA. The viremia of PUUV is known to disappear soon after the first week, which was also seen in the samples of two patients that were RNA negative but IgM positive. For this very reason, serology will be needed for a reliable diagnosis of NE. Based on our results, we, however, suggest that PUUV-qRT-PCR could be a complementary method beside serology and used in routine diagnostics to detect PUUV and diagnose NE in an early phase of the infection in particular, as we diagnosed acute NE in one patient first by PUUV-qRT-PCR, and only later by serology. Furthermore, PUUV-qRT-PCR has been shown to be robust and rather insensitive to inhibitors in different sample types [22] and it is also useful for post-mortem analysis (our unpublished data). This method could also be used for measuring viral load, which has been shown to correlate with disease severity for other hantaviruses [23,24]. For PUUV infections, the correlation seems to be lacking, while other markers for disease severity have already been suggested [15,25].

The sequence analysis together with PUUV IgM positive samples originating from different parts of Finland confirms that this method detects all PUUV variants circulating in Finland. As seen in Figure 1, all Finnish PUUV variants are monophyletic, and no geographical clustering is observed. Further studies, and most likely optimization of primers and probe, are needed to see if the method detects strains circulating outside Finland.

## Figures and Tables

**Table 1:** Comparison of Puumala virus (PUUV) antibody and RNA detection.

Patient No	Sex	Age	Hospital District	IgM	PUUV-qRT-PCR	RT-Nested-PCR
1	F	39	HUS	pos	pos	pos
2	M	25	HUS	pos	pos	**NEG**
3	F	38	HUS	pos	pos	pos
4	M	55	HUS	pos	**NEG**	**NEG**
5	F	45	HUS	pos	**NEG**	**NEG**
6	M	65	HUS	pos	pos	pos
7	F	30	HUS	pos	pos	**NEG**
8	F	61	LP	pos	pos	pos
9	F	26	HUS	pos	pos	pos
10	M	57	HUS	pos	pos	pos
11	F	47	HUS	pos	pos	pos
12	F	64	HUS	pos	pos	pos
13	M	29	KYM	pos	pos	pos
14	F	61	HUS	pos	pos	pos
15	F	32	HUS	pos	pos	pos
16	M	46	HUS	pos	pos	pos
17	F	65	KYM	pos	pos	pos
18	M	66	HUS	pos	pos	**NEG**
19	M	71	SO	pos	pos	pos
20	M	77	TP	pos	pos	pos
21	M	48	CF	pos	pos	pos
22	F	28	HUS	pos	pos	pos
23	M	50	HUS	pos	pos	pos
24	M	39	HUS	pos	pos	pos
25	M	51	CF	pos	pos	pos
26	F	66	HUS	pos	pos	pos
27	F	51	HUS	pos	pos	pos
28	M	43	TP	pos	pos	pos
29	M	54	HUS	pos	pos	pos
30	M	57	HUS	pos	pos	pos
No of positives:				**30**	**28**	**25**

PUUV, Puumala virus; F, female; M, male; IgM, immunoglobulin M; No, number; pos, positive; neg, negative. HUS, Helsinki and Uusimaa; LP, Länsi-Pohja; KYM, Kymenlaakso; SO, Southern Ostrobothnia; TP, Tavastia Proper; CF, Central Finland.
